# Synthesis and structure of (*Z*)-2-(eth­oxy­methyl­idene)-8-methyl-2,3,4,9-tetra­hydro-1*H*-carbazol-1-one

**DOI:** 10.1107/S2056989026005049

**Published:** 2026-05-15

**Authors:** Makuteswaran Sridharan, Aravazhi Amalan Thiruvalluvar

**Affiliations:** a Department of Chemistry, RV College of Engineering, Bangalore 560 059, Karnataka, India; b Principal (Retired), 63 Shanthi Nagar, 5th Street, Nanjikottai Road, Thanjavur 613 006, Tamilnadu, India; University of Aberdeen, United Kingdom

**Keywords:** crystal structure, carbazol-1-one, Hirshfeld surface, energy framework analysis

## Abstract

In the title compound, C_16_H_17_NO_2_, the cyclo­hexene ring adopts an envelope conformation and the side chain is extended. In the extended structure, N—H⋯O hydrogen bonds connect the mol­ecules into [100] chains and weak C—H⋯O inter­actions and pairwise C—H⋯π inter­actions consolidate the packing. A Hirshfeld surface analysis indicates that the most important contributions to the crystal packing are from H⋯H (57.6%), C⋯H/H⋯C (24.2%), and H⋯O/ O⋯H (13.8%) contacts. Evaluation of the electrostatic, dispersion and total energy frameworks indicates that the cohesion of the crystal largely depends on dispersion energy contributions.

## Abstract 2

In the extended structure of the title compound, N—H⋯O hydrogen bonds connect the mol­ecules into [100] chains and weak C—H⋯O inter­actions and pairwise C—H⋯π inter­actions consolidate the packing.

## 1. Chemical context

Carbazole derivatives represent a class of heteroaromatic compounds that continue to inspire extensive investigation in both organic synthesis and medicinal chemistry (Knölker & Reddy, 2002 ▸). Substitution at different positions of the carbazole ring system has yielded derivatives with enhanced reactivity and diverse biological properties, underscoring the versatility of this structural motif.

Within this context, 2,3,4,9-tetra­hydro­carbazol-1-ones have proven to be valuable precursors, offering a convenient entry point to more elaborate heterocycles. Of particular inter­est is 2-(eth­oxy­methyl­ene)-2,3,4,9-tetra­hydro­carbazol-1-one, which has been reported as a versatile inter­mediate in heterocyclic synthesis (Sridharan & Thiruvalluar, 2026 ▸). The eth­oxy­methyl­ene substituent (Dasgupta & Ghatak, 1985 ▸) at the 2-position introduces an electrophilic site amenable to condensation and cyclization, while the carbonyl group at the 1-position enhances synthetic flexibility. This dual functionality renders the compound an effective building block for the construction of complex heterocycles with potential pharmaceutical relevance.
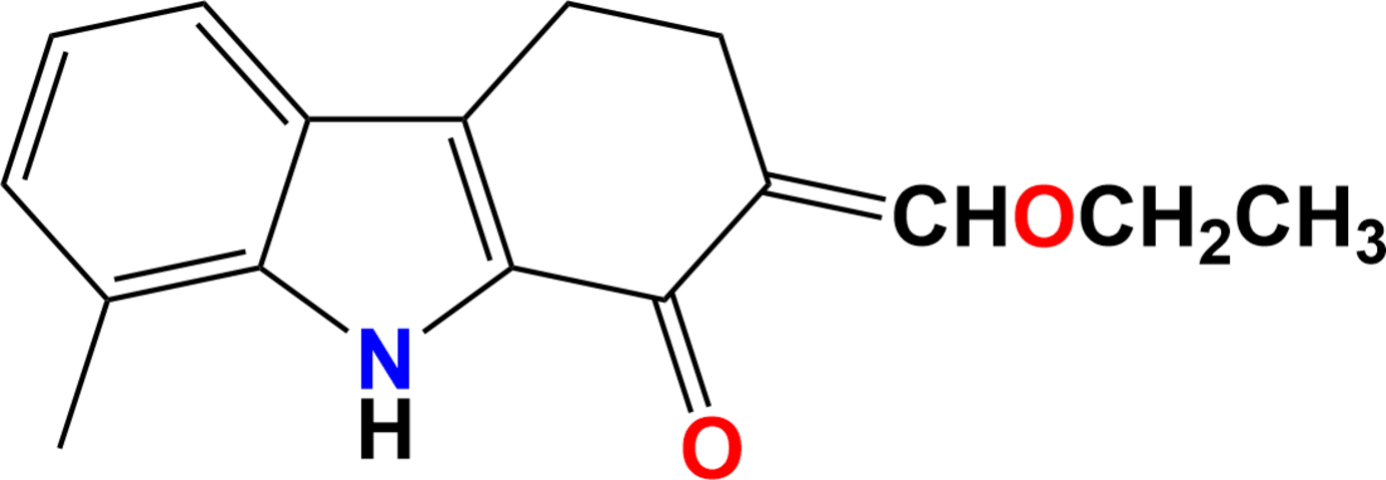


As part of our studies in this area, we now describe the synthesis and structure of the title compound, C_16_H_17_NO_2_ (**I**).

## 2. Structural commentary

**Figure 1:**
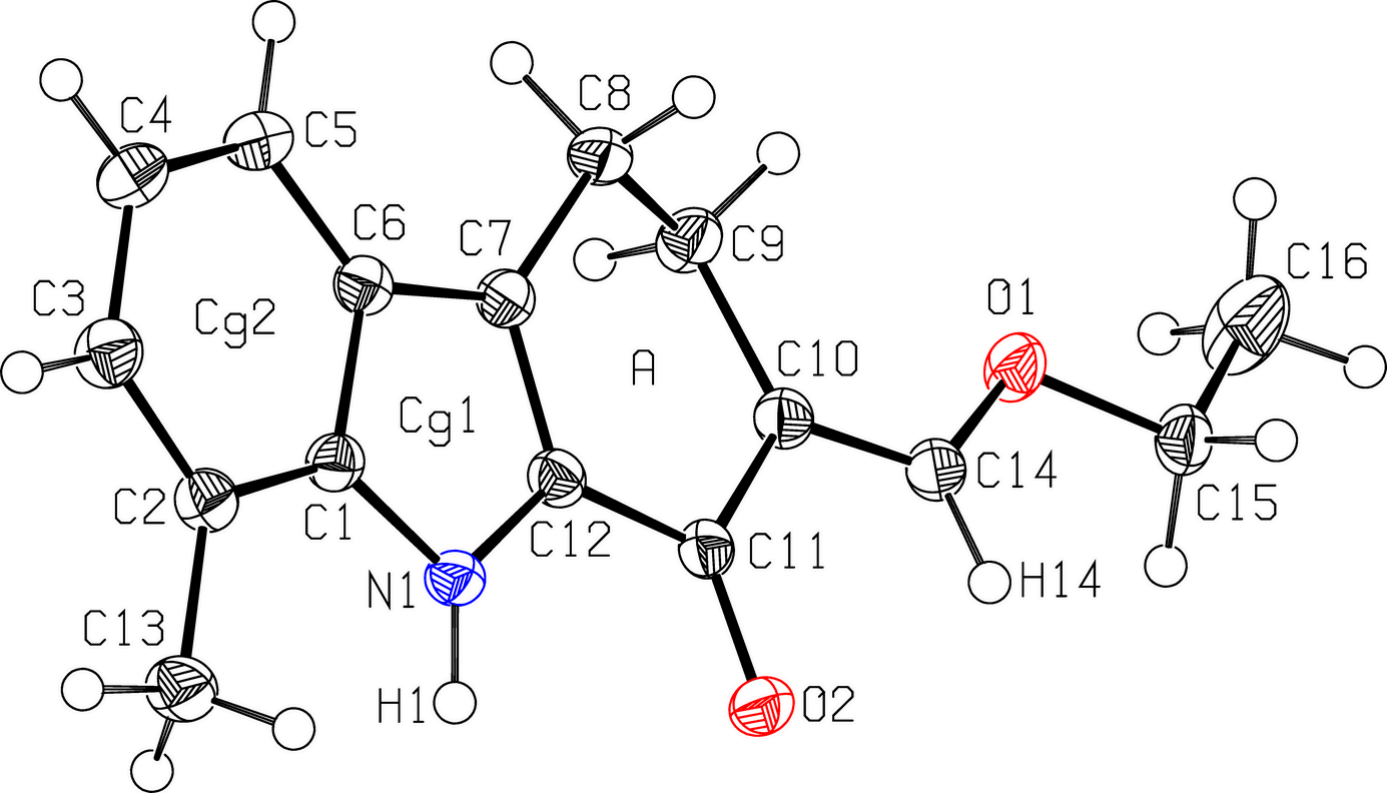
The mol­ecular structure of (**I**), showing displacement ellipsoids drawn at the 50% probability level.

As shown in Fig. 1 ▸, compound (**I**), which crystallizes in the ortho­rhom­bic space group *Pbca* with one mol­ecule in the asymmetric unit, consists of indole and cyclo­hexene units fused *via* the C7—C12 bond. As expected, the pyrrole (N1/C1/C6/C7/C12) and benzene (C1–C6) rings are nearly co-planar, subtending a dihedral angle of 1.38 (6)°. A puckering analysis (Cremer & Pople, 1975 ▸) of the C7–C12 ring gave the parameters: *q*_2_ = 0.3271 (13) Å, *q*_3_ = −0.1784 (13) Å, *Q*_T_ = 0.3726 (13) Å, θ = 118.6 (2)° and φ = 280.9 (2)°, which corresponds to an envelope conformation, where atom C9 is at the flap position and displaced by −0.497 (2) Å from the best plane of the remaining tricyclic carbazole non-H atoms. The C7—C8—C9—C10 torsion angle is −42.40 (15)°. The eth­oxy­methyl­ene side chain adopts an extended conformation, as indicated by the C10—C14—O1—C15 and C14—O1—C15—C16 torsion angles of 176.41 (11) and 174.05 (13)°, respectively.

## 3. Supra­molecular features

**Figure 2:**
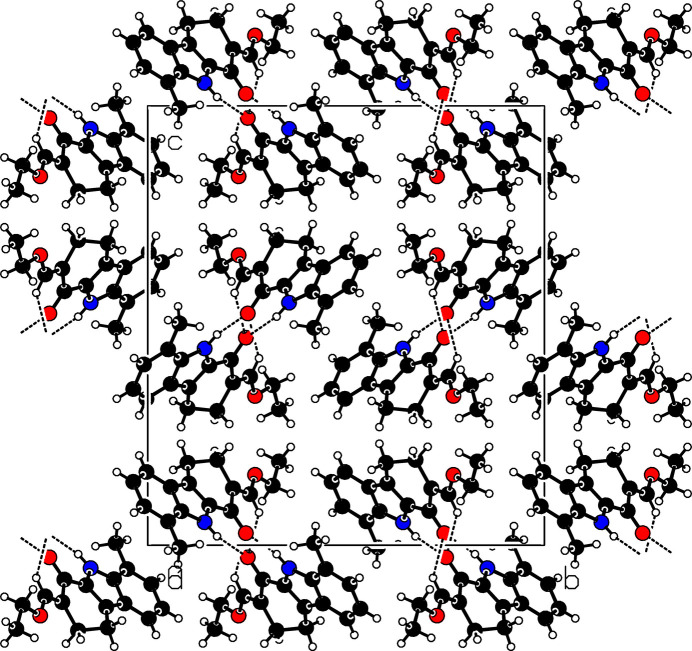
Partial packing view of (**I**), viewed down the *a-*axis direction, showing the hydrogen bonds. Black dashed lines represent C—H⋯O and N—H⋯O hydrogen bonds.

**Figure 3:**
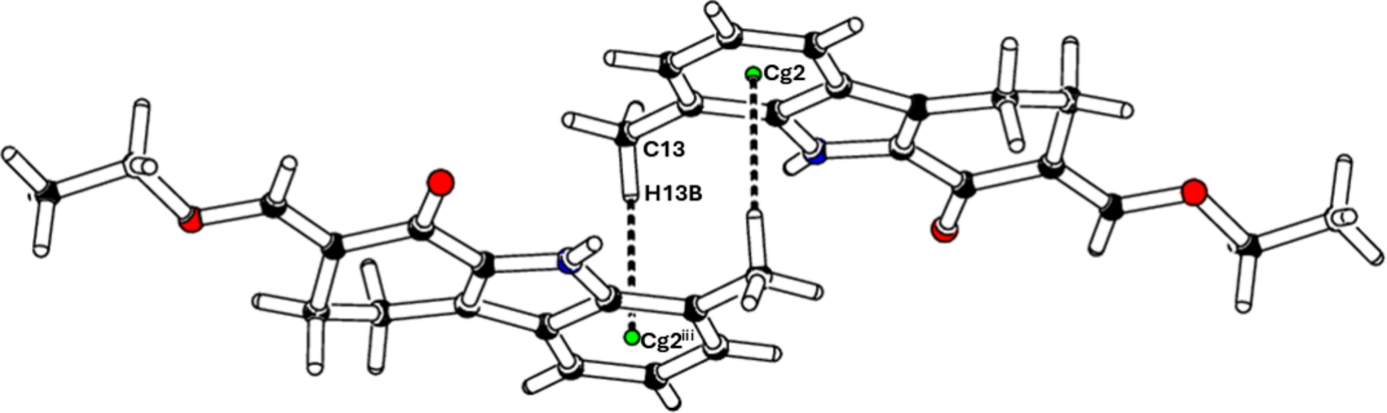
Straw-style packing view of (**I**), showing the C—H⋯π contacts. Centroids are shown as green spheres and black dashed lines are H⋯π contacts.

In the extended structure, strong N1—H1⋯O2 hydrogen bonds (Table 1 ▸) form *C*(7) chains of mol­ecules propagating parallel to the *a-*axis direction as shown in Fig. 2 ▸. Weak C14—H14⋯O2 links generate double chains. The mol­ecules are further linked by pairwise C13—H13*B*⋯*Cg*2 (where *Cg*2 is the centroid of the C1–C6 benzene ring) inter­actions that connect parallel chains with each other (Fig. 3 ▸). No significant π–π stacking inter­actions are observed in this structure.

## 4. Database survey

A search of the Cambridge Structural Database (CSD, Version 6.01, updated to February 2026; Groom *et al.*, 2016 ▸) using the core structure of (**I**) gave zero hits.

## 5. Hirshfeld surface

**Figure 4:**
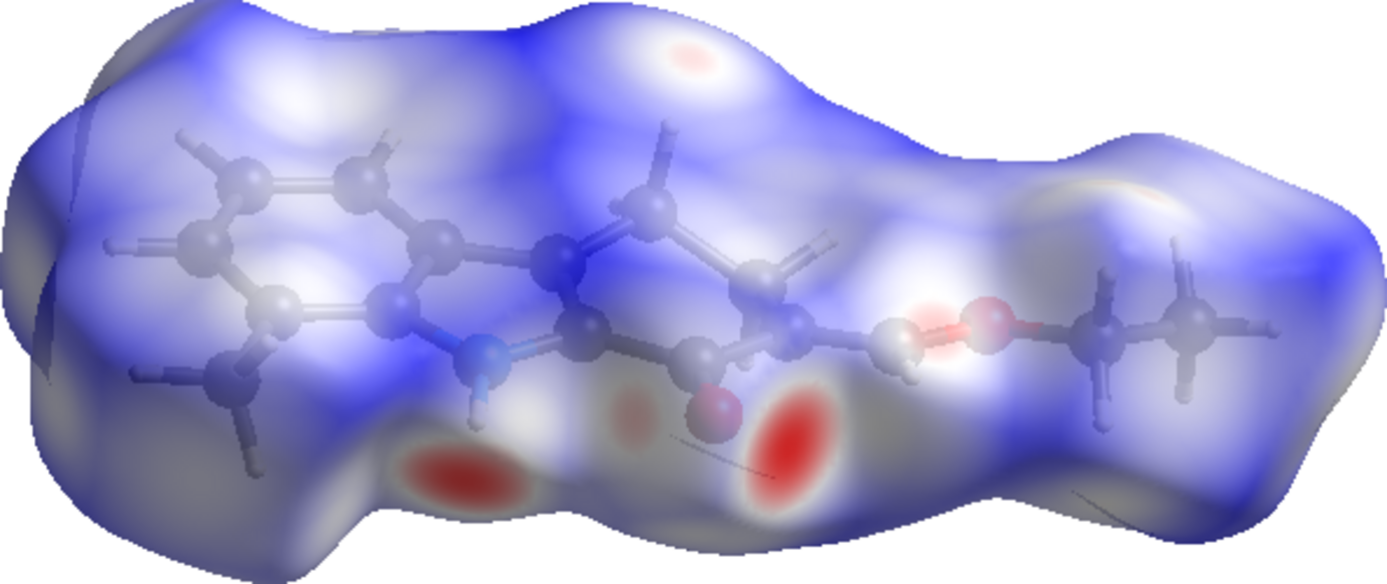
View of the three-dimensional Hirshfeld surface of (**I**), plotted over *d*_norm_ in the range from −0.51 to 1.22 a.u. with a neighbouring mol­ecule.

**Figure 5:**
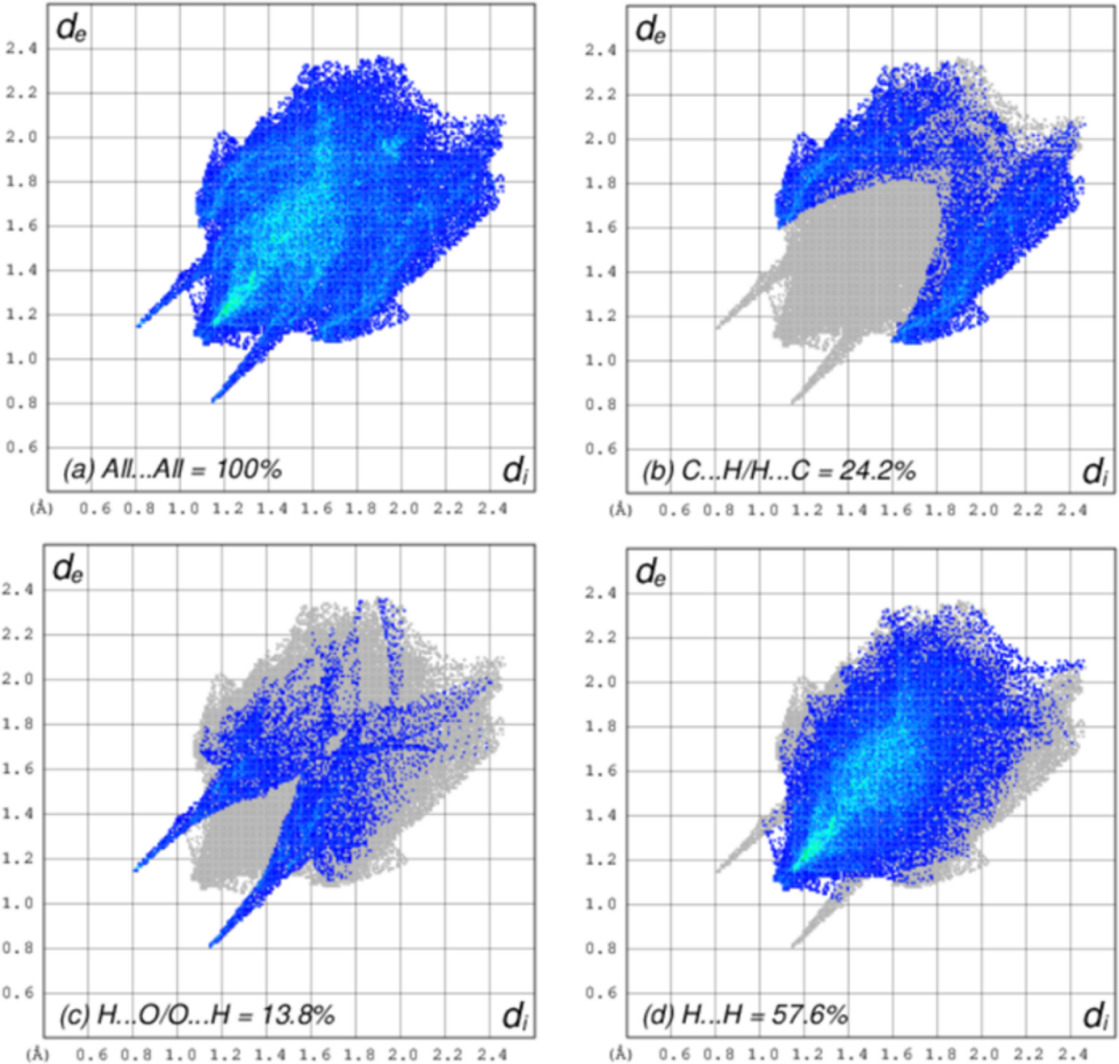
Two-dimensional fingerprint plots for (**I**), showing (*a*) all inter­actions, and those showing (*b*) C⋯H/H⋯C, (*c*) H⋯O/O⋯H, and (*d*) H⋯H inter­actions. The *d*_i_ and *d*_e_ values are the closest inter­nal and external distances (in Å) from given points on the Hirshfeld surface.

A Hirshfeld surface (HS) analysis was carried out using *CrystalExplorer* version 21.5 (Spackman *et al.*, 2021 ▸) to further qu­antify the inter­molecular inter­actions in the crystal of (**I**). The HS plotted over *d*_norm_ is shown in Fig. 4 ▸, where the bright-red spots correspond to donor and/or acceptor sites. According to the two-dimensional fingerprint plots (Fig. 5 ▸), C⋯H/H⋯C, H⋯O/O⋯H and H⋯H contacts make the most important contributions to the HS, with values of 24.2%, 13.8%, and 57.6% respectively. All other contact types, including C⋯N/N⋯C, C⋯O/O⋯C, H⋯N/N⋯H, N⋯O/O⋯N, and C⋯C contribute less than 2.0% each to the total.

## 6. Inter­action energy calculations and energy frameworks

**Figure 6:**
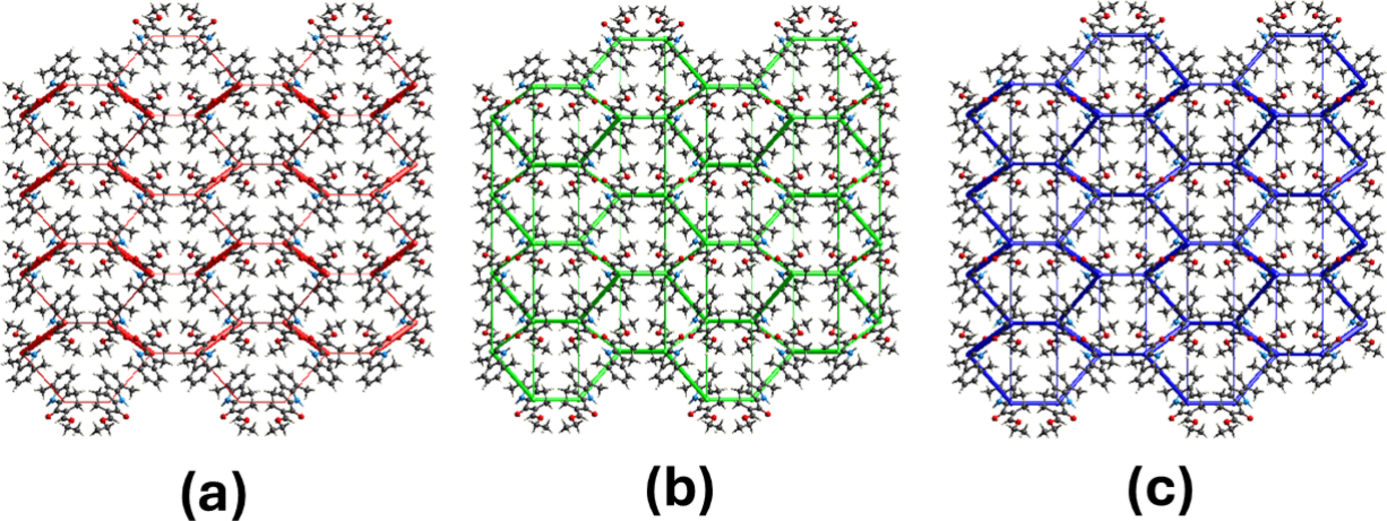
The energy frameworks for a cluster of mol­ecules of (**I**) viewed down the *a-*axis direction, showing (*a*) electrostatic energy *E*_ele_, (*b*) dispersion energy *E*_dis_ and (*c*) total energy *E*_tot_ diagrams. The cylindrical radius is proportional to the relative strength of the corresponding energies and they were adjusted to the same scale factor of 80 with a cut-off value of 5 kJ mol^−1^ within 2 × 2 × 2 unit cells.

The CE-B3LYP/6-31G(d,p) energy model available in *CrystalExplorer* was used to calculate the inter­molecular inter­action energies. Hydrogen-bonding inter­action energies (in kJ mol^−1^) were calculated to be −41.4 (*E*_ele_), −13.9 (*E*_pol_), −23.1 (*E*_dis_), 45.3 (*E*_rep_) and −46.3 (*E*_tot_) for the collective hydrogen bonds N1—H1⋯O2 and C14—H14⋯O2. Values of −9.4 (*E*_ele_), −1.8 (*E*_pol_), −38.8 (*E*_dis_), 21.6 (*E*_rep_) and −31.7 (*E*_tot_) arose for the C13—H13*B*⋯π inter­action. Energy frameworks (Turner *et al.*, 2015 ▸) were constructed for *E*_ele_ (red cylinders), *E*_dis_ (green cylinders) and *E*_tot_ (blue cylinders) [Fig. 6 ▸(*a*)–(*c*)], and their evaluation indicates that crystal cohesion largely depends on dispersion energy contributions in the crystal structure of (**I**).

## 7. Synthesis and crystallization

**Figure 7:**
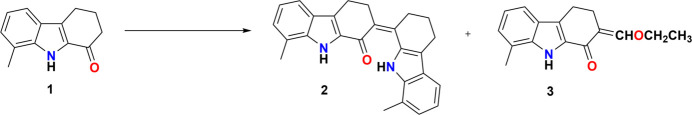
The synthesis scheme for (**I**).

8-Methyl-2,3,4,9-tetra­hydro­carbazol-1-one (**1**) (1.00 g, 0.005 mol) in di­chloro­methane (15 ml) was added to an ice-cooled solution of di­eth­oxy­carbenium fluoroborate [prepared *in situ* from 1.65 ml of BF_3_·Et_2_O (0.01 mol) and 1.25 ml of HC(OEt_3_) (0.01 mol)]. The reaction mixture (Fig. 7 ▸) was kept at 258 to 263 K. To this mixture, tri­ethyl­amine (0.01 mol) was added dropwise and the stirring was continued over a period of five h. The reaction was monitored by TLC. After the completion of reaction, the excess solvent was removed and extracted using ethyl acetate dried over anhydrous sodium sulfate. The brown solid separated out was then separated by column chromatography over silica gel using petroleum ether: ethyl acetate as eluants (99:1) and (95:5) to yield (*Z*)-8-methyl-2,3,4,9-tetra­hydro-2 (8′-methyl-2′,3′,4′,9′-tetra­hydro­carbazol-1-yl­idene)-carbazol 1-one (**2**) and (*Z*)-2-(eth­oxy­methyl­ene)-8-methyl-2,3,4,9 tetra­hydro-1*H*-carbazol-1-one (**3**), respectively. The chemical structure of the final products was confirmed by NMR spectroscopy and elementary analysis data. Compound (**3**) was recrystallized from ethanol solution as yellow prisms (0.842 g, 66%), m.p. 377–379 K. The reaction scheme is shown in Fig. 7 ▸.

## 8. Refinement

Crystal data, data collection and structure refinement details are summarized in Table 2 ▸. The N-bound H atom was located in a difference-Fourier map and its position was freely refined with *U*_iso_(H) = 1.2*U*_eq_(N). All the other H atoms were placed in calculated positions and refined as riding atoms with *U*_iso_(H) = 1.2*U*_eq_(C) or 1.5*U*_eq_(methyl C). The methyl group was allowed to rotate, but not to tip, to best fit the experimental electron density.

## Supplementary Material

Crystal structure: contains datablock(s) I. DOI: 10.1107/S2056989026005049/hb8217sup1.cif

Structure factors: contains datablock(s) I. DOI: 10.1107/S2056989026005049/hb8217Isup2.hkl

Supporting information file. DOI: 10.1107/S2056989026005049/hb8217Isup3.cdx

Supporting information file. DOI: 10.1107/S2056989026005049/hb8217Isup4.cml

CCDC reference: 1540675

Additional supporting information:  crystallographic information; 3D view; checkCIF report

## Figures and Tables

**Table 1:** Hydrogen-bond geometry (Å, °) *Cg*2 is the centroid of the benzene (C1–C6) ring.

*D*—H⋯*A*	*D*—H	H⋯*A*	*D*⋯*A*	*D*—H⋯*A*
N1—H1⋯O2^i^	0.919 (17)	2.034 (17)	2.9235 (14)	162.2 (15)
C14—H14⋯O2^ii^	0.95	2.52	3.2396 (15)	133
C13—H13*B*⋯*Cg*2^iii^	0.98	2.76	3.5669 (15)	140

Symmetry codes: (i) 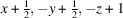; (ii) 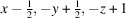; (iii) 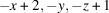.

**Table 2:** Experimental details

Crystal data	
Chemical formula	C_16_H_17_NO_2_
*M*_r_	255.30
Crystal system, space group	Orthorhombic, *Pbca*
Temperature (K)	100
*a*, *b*, *c* (Å)	6.9872 (5), 18.3913 (13), 20.3450 (15)
*V* (Å^3^)	2614.4 (3)
*Z*	8
Radiation type	Mo *K*α
μ (mm^−1^)	0.09
Crystal size (mm)	0.68 × 0.46 × 0.43

Data collection	
Diffractometer	Bruker AXS SMART APEX CCD
Absorption correction	Multi-scan (*SADABS2004*; Krause *et al.*, 2015 ▸)
*T*_min_, *T*_max_	0.885, 0.964
No. of measured, independent and observed [*I* > 2σ(*I*)] reflections	18986, 3238, 2854
*R*_int_	0.029
(sin θ/λ)_max_ (Å^−1^)	0.667

Refinement	
*R*[*F*^2^ > 2σ(*F*^2^)], *wR*(*F*^2^), *S*	0.046, 0.124, 1.04
No. of reflections	3238
No. of parameters	177
H-atom treatment	H atoms treated by a mixture of independent and constrained refinement
Δρ_max_, Δρ_min_ (e Å^−3^)	0.35, −0.31

Computer programs: *SMART* (Bruker, 2002 ▸), *SAINT-Plus* (Bruker, 2003 ▸), *SHELXS* (Sheldrick, 2008 ▸), *SHELXL2025/1* (Sheldrick, 2015 ▸), *ORTEP-3 for Windows* (Farrugia, 2012 ▸), *PLATON* (Spek, 2020 ▸) and *publCIF* (Westrip, 2010 ▸).

## supplementary crystallographic information

### (Z)-2-(Ethoxymethylidene)-8-methyl-2,3,4,9-tetrahydro-1H-carbazol-1-one Crystal data

**Table:**

C_16_H_17_NO_2_	*D*_x_ = 1.297 Mg m^−^^3^
*M**_r_* = 255.30	Mo *K*α radiation, λ = 0.71073 Å
Orthorhombic, *P**b**c**a*	Cell parameters from 6074 reflections
*a* = 6.9872 (5) Å	θ = 2.4–30.4°
*b* = 18.3913 (13) Å	µ = 0.09 mm^−^^1^
*c* = 20.3450 (15) Å	*T* = 100 K
*V* = 2614.4 (3) Å^3^	Block, yellow
*Z* = 8	0.68 × 0.46 × 0.43 mm
*F*(000) = 1088	

### (Z)-2-(Ethoxymethylidene)-8-methyl-2,3,4,9-tetrahydro-1H-carbazol-1-one Data collection

**Table:**

Bruker AXS SMART APEX CCD diffractometer	3238 independent reflections
Radiation source: fine-focus sealed tube	2854 reflections with *I* > 2σ(*I*)
Graphite monochromator	*R*_int_ = 0.029
ω scans	θ_max_ = 28.3°, θ_min_ = 2.2°
Absorption correction: multi-scan (*SADABS2004*; Krause *et al.*, 2015)	*h* = −9→9
*T*_min_ = 0.885, *T*_max_ = 0.964	*k* = −23→24
18986 measured reflections	*l* = −23→27

### (Z)-2-(Ethoxymethylidene)-8-methyl-2,3,4,9-tetrahydro-1H-carbazol-1-one Refinement

**Table:**

Refinement on *F*^2^	Primary atom site location: structure-invariant direct methods
Least-squares matrix: full	Secondary atom site location: difference Fourier map
*R*[*F*^2^ > 2σ(*F*^2^)] = 0.046	Hydrogen site location: mixed
*wR*(*F*^2^) = 0.124	H atoms treated by a mixture of independent and constrained refinement
*S* = 1.04	*w* = 1/[σ^2^(*F*_o_^2^) + (0.0659*P*)^2^ + 1.1726*P*] where *P* = (*F*_o_^2^ + 2*F*_c_^2^)/3
3238 reflections	(Δ/σ)_max_ < 0.001
177 parameters	Δρ_max_ = 0.35 e Å^−^^3^
0 restraints	Δρ_min_ = −0.31 e Å^−^^3^

### (Z)-2-(Ethoxymethylidene)-8-methyl-2,3,4,9-tetrahydro-1H-carbazol-1-one Special details

**Table:**

Geometry. All esds (except the esd in the dihedral angle between two l.s. planes) are estimated using the full covariance matrix. The cell esds are taken into account individually in the estimation of esds in distances, angles and torsion angles; correlations between esds in cell parameters are only used when they are defined by crystal symmetry. An approximate (isotropic) treatment of cell esds is used for estimating esds involving l.s. planes.

### (Z)-2-(Ethoxymethylidene)-8-methyl-2,3,4,9-tetrahydro-1H-carbazol-1-one Fractional atomic coordinates and isotropic or equivalent isotropic displacement parameters (Å^2^)

**Table:**

	*x*	*y*	*z*	*U*_iso_*/*U*_eq_	
C1	0.98167 (17)	0.08476 (6)	0.42264 (6)	0.0194 (2)	
C2	1.14974 (18)	0.05163 (7)	0.44576 (6)	0.0225 (3)	
C3	1.20971 (19)	−0.00928 (7)	0.41145 (7)	0.0264 (3)	
H3	1.321352	−0.034028	0.425941	0.032*	
C4	1.11146 (19)	−0.03616 (7)	0.35569 (6)	0.0264 (3)	
H4	1.159354	−0.077761	0.333422	0.032*	
C5	0.94764 (18)	−0.00293 (6)	0.33325 (6)	0.0229 (3)	
H5	0.882376	−0.020940	0.295664	0.027*	
C6	0.87898 (17)	0.05835 (6)	0.36728 (6)	0.0198 (2)	
C7	0.71601 (17)	0.10427 (6)	0.35948 (6)	0.0197 (2)	
C8	0.55199 (18)	0.10052 (7)	0.31249 (6)	0.0235 (3)	
H8A	0.457131	0.064632	0.328424	0.028*	
H8B	0.598480	0.084221	0.268949	0.028*	
C9	0.45628 (18)	0.17532 (7)	0.30579 (6)	0.0258 (3)	
H9A	0.534229	0.205797	0.275922	0.031*	
H9B	0.328899	0.168899	0.285286	0.031*	
C10	0.43179 (17)	0.21508 (7)	0.37046 (6)	0.0215 (3)	
C11	0.57990 (17)	0.20979 (7)	0.42188 (6)	0.0201 (2)	
C12	0.72546 (16)	0.15533 (6)	0.40907 (6)	0.0198 (2)	
C13	1.2535 (2)	0.08035 (7)	0.50504 (7)	0.0276 (3)	
H13A	1.369795	0.051699	0.512380	0.041*	
H13B	1.170246	0.076726	0.543665	0.041*	
H13C	1.288073	0.131352	0.497714	0.041*	
C14	0.28156 (17)	0.25820 (7)	0.38372 (6)	0.0222 (3)	
H14	0.273853	0.280696	0.425672	0.027*	
C15	−0.01415 (17)	0.31500 (7)	0.36423 (7)	0.0251 (3)	
H15A	0.036691	0.360927	0.382643	0.030*	
H15B	−0.082869	0.288797	0.399579	0.030*	
C16	−0.1470 (3)	0.33099 (11)	0.30927 (8)	0.0480 (5)	
H16A	−0.081211	0.360702	0.276282	0.072*	
H16B	−0.258376	0.357541	0.326052	0.072*	
H16C	−0.189264	0.285313	0.289162	0.072*	
N1	0.88530 (14)	0.14364 (6)	0.44784 (5)	0.0202 (2)	
O1	0.14156 (13)	0.27071 (5)	0.33973 (4)	0.0259 (2)	
O2	0.58335 (13)	0.24760 (5)	0.47242 (4)	0.0253 (2)	
H1	0.929 (2)	0.1750 (9)	0.4797 (8)	0.030*	

### (Z)-2-(Ethoxymethylidene)-8-methyl-2,3,4,9-tetrahydro-1H-carbazol-1-one Atomic displacement parameters (Å^2^)

**Table:**

	*U*^11^	*U*^22^	*U*^33^	*U*^12^	*U*^13^	*U*^23^
C1	0.0206 (5)	0.0199 (5)	0.0178 (5)	−0.0011 (4)	0.0012 (4)	0.0003 (4)
C2	0.0223 (6)	0.0235 (6)	0.0216 (6)	0.0001 (4)	−0.0012 (5)	0.0012 (5)
C3	0.0254 (6)	0.0258 (6)	0.0278 (6)	0.0049 (5)	−0.0019 (5)	−0.0001 (5)
C4	0.0314 (7)	0.0223 (6)	0.0254 (6)	0.0038 (5)	0.0011 (5)	−0.0024 (5)
C5	0.0289 (6)	0.0201 (6)	0.0196 (6)	−0.0011 (5)	−0.0001 (5)	−0.0008 (4)
C6	0.0207 (5)	0.0204 (5)	0.0181 (5)	−0.0021 (4)	0.0004 (4)	0.0017 (4)
C7	0.0200 (5)	0.0214 (5)	0.0178 (5)	−0.0016 (4)	0.0004 (4)	0.0004 (4)
C8	0.0221 (6)	0.0281 (6)	0.0202 (6)	0.0002 (5)	−0.0034 (5)	−0.0041 (5)
C9	0.0241 (6)	0.0340 (7)	0.0192 (6)	0.0062 (5)	−0.0035 (5)	−0.0039 (5)
C10	0.0194 (5)	0.0267 (6)	0.0184 (5)	−0.0006 (4)	−0.0009 (4)	−0.0007 (4)
C11	0.0179 (5)	0.0231 (6)	0.0192 (5)	−0.0020 (4)	0.0013 (4)	−0.0006 (4)
C12	0.0182 (5)	0.0229 (6)	0.0184 (5)	−0.0014 (4)	−0.0012 (4)	0.0003 (4)
C13	0.0254 (6)	0.0305 (6)	0.0269 (6)	0.0028 (5)	−0.0066 (5)	−0.0022 (5)
C14	0.0201 (6)	0.0280 (6)	0.0185 (5)	−0.0006 (5)	−0.0004 (4)	−0.0006 (4)
C15	0.0196 (6)	0.0263 (6)	0.0293 (7)	0.0030 (5)	0.0029 (5)	−0.0009 (5)
C16	0.0433 (9)	0.0655 (11)	0.0353 (8)	0.0311 (8)	−0.0068 (7)	−0.0040 (8)
N1	0.0193 (5)	0.0219 (5)	0.0195 (5)	0.0009 (4)	−0.0025 (4)	−0.0026 (4)
O1	0.0207 (4)	0.0350 (5)	0.0221 (4)	0.0076 (4)	−0.0023 (3)	−0.0034 (4)
O2	0.0229 (4)	0.0302 (5)	0.0227 (4)	0.0031 (4)	−0.0023 (3)	−0.0075 (4)

### (Z)-2-(Ethoxymethylidene)-8-methyl-2,3,4,9-tetrahydro-1H-carbazol-1-one Geometric parameters (Å, º)

**Table:**

C1—N1	1.3744 (15)	C9—H9B	0.9900
C1—C2	1.4041 (17)	C10—C14	1.3430 (17)
C1—C6	1.4211 (16)	C10—C11	1.4747 (17)
C2—C3	1.3848 (18)	C11—O2	1.2416 (15)
C2—C13	1.5030 (17)	C11—C12	1.4510 (16)
C3—C4	1.4152 (18)	C12—N1	1.3841 (15)
C3—H3	0.9500	C13—H13A	0.9800
C4—C5	1.3755 (18)	C13—H13B	0.9800
C4—H4	0.9500	C13—H13C	0.9800
C5—C6	1.4069 (17)	C14—O1	1.3457 (15)
C5—H5	0.9500	C14—H14	0.9500
C6—C7	1.4266 (16)	C15—O1	1.4476 (15)
C7—C12	1.3799 (16)	C15—C16	1.483 (2)
C7—C8	1.4940 (16)	C15—H15A	0.9900
C8—C9	1.5356 (18)	C15—H15B	0.9900
C8—H8A	0.9900	C16—H16A	0.9800
C8—H8B	0.9900	C16—H16B	0.9800
C9—C10	1.5150 (17)	C16—H16C	0.9800
C9—H9A	0.9900	N1—H1	0.919 (17)
			
N1—C1—C2	128.81 (11)	C14—C10—C9	123.21 (11)
N1—C1—C6	108.52 (10)	C11—C10—C9	120.33 (11)
C2—C1—C6	122.65 (11)	O2—C11—C12	121.45 (11)
C3—C2—C1	115.81 (11)	O2—C11—C10	124.35 (11)
C3—C2—C13	122.87 (11)	C12—C11—C10	114.20 (10)
C1—C2—C13	121.31 (11)	C7—C12—N1	110.46 (10)
C2—C3—C4	122.67 (12)	C7—C12—C11	124.58 (11)
C2—C3—H3	118.7	N1—C12—C11	124.78 (11)
C4—C3—H3	118.7	C2—C13—H13A	109.5
C5—C4—C3	120.96 (12)	C2—C13—H13B	109.5
C5—C4—H4	119.5	H13A—C13—H13B	109.5
C3—C4—H4	119.5	C2—C13—H13C	109.5
C4—C5—C6	118.45 (12)	H13A—C13—H13C	109.5
C4—C5—H5	120.8	H13B—C13—H13C	109.5
C6—C5—H5	120.8	C10—C14—O1	122.38 (11)
C5—C6—C1	119.45 (11)	C10—C14—H14	118.8
C5—C6—C7	133.76 (11)	O1—C14—H14	118.8
C1—C6—C7	106.79 (10)	O1—C15—C16	108.82 (11)
C12—C7—C6	106.46 (10)	O1—C15—H15A	109.9
C12—C7—C8	122.41 (11)	C16—C15—H15A	109.9
C6—C7—C8	131.01 (11)	O1—C15—H15B	109.9
C7—C8—C9	110.46 (10)	C16—C15—H15B	109.9
C7—C8—H8A	109.6	H15A—C15—H15B	108.3
C9—C8—H8A	109.6	C15—C16—H16A	109.5
C7—C8—H8B	109.6	C15—C16—H16B	109.5
C9—C8—H8B	109.6	H16A—C16—H16B	109.5
H8A—C8—H8B	108.1	C15—C16—H16C	109.5
C10—C9—C8	113.86 (10)	H16A—C16—H16C	109.5
C10—C9—H9A	108.8	H16B—C16—H16C	109.5
C8—C9—H9A	108.8	C1—N1—C12	107.77 (10)
C10—C9—H9B	108.8	C1—N1—H1	126.4 (10)
C8—C9—H9B	108.8	C12—N1—H1	124.9 (10)
H9A—C9—H9B	107.7	C14—O1—C15	114.43 (10)
C14—C10—C11	116.42 (11)		
			
N1—C1—C2—C3	177.44 (12)	C8—C9—C10—C11	36.83 (16)
C6—C1—C2—C3	−0.47 (18)	C14—C10—C11—O2	−7.56 (19)
N1—C1—C2—C13	−1.2 (2)	C9—C10—C11—O2	170.33 (12)
C6—C1—C2—C13	−179.16 (11)	C14—C10—C11—C12	172.13 (11)
C1—C2—C3—C4	1.28 (19)	C9—C10—C11—C12	−9.99 (16)
C13—C2—C3—C4	179.95 (13)	C6—C7—C12—N1	−0.32 (13)
C2—C3—C4—C5	−0.9 (2)	C8—C7—C12—N1	176.05 (11)
C3—C4—C5—C6	−0.35 (19)	C6—C7—C12—C11	−175.65 (11)
C4—C5—C6—C1	1.11 (17)	C8—C7—C12—C11	0.72 (19)
C4—C5—C6—C7	−178.20 (13)	O2—C11—C12—C7	169.91 (12)
N1—C1—C6—C5	−179.00 (10)	C10—C11—C12—C7	−9.78 (17)
C2—C1—C6—C5	−0.72 (18)	O2—C11—C12—N1	−4.76 (19)
N1—C1—C6—C7	0.48 (13)	C10—C11—C12—N1	175.55 (11)
C2—C1—C6—C7	178.76 (11)	C11—C10—C14—O1	176.51 (11)
C5—C6—C7—C12	179.28 (13)	C9—C10—C14—O1	−1.3 (2)
C1—C6—C7—C12	−0.09 (13)	C2—C1—N1—C12	−178.82 (12)
C5—C6—C7—C8	3.3 (2)	C6—C1—N1—C12	−0.68 (13)
C1—C6—C7—C8	−176.04 (12)	C7—C12—N1—C1	0.63 (13)
C12—C7—C8—C9	25.96 (16)	C11—C12—N1—C1	175.94 (11)
C6—C7—C8—C9	−158.65 (12)	C10—C14—O1—C15	176.41 (11)
C7—C8—C9—C10	−42.40 (15)	C16—C15—O1—C14	174.05 (13)
C8—C9—C10—C14	−145.43 (12)		

### (Z)-2-(Ethoxymethylidene)-8-methyl-2,3,4,9-tetrahydro-1H-carbazol-1-one Hydrogen-bond geometry (Å, º)

*Cg*2 is the centroid of the benzene (C1–C6) ring.

**Table:**

*D*—H···*A*	*D*—H	H···*A*	*D*···*A*	*D*—H···*A*
N1—H1···O2^i^	0.919 (17)	2.034 (17)	2.9235 (14)	162.2 (15)
C14—H14···O2^ii^	0.95	2.52	3.2396 (15)	133
C13—H13*B*···*Cg*2^iii^	0.98	2.76	3.5669 (15)	140

Symmetry codes: (i) *x*+1/2, −*y*+1/2, −*z*+1; (ii) *x*−1/2, −*y*+1/2, −*z*+1; (iii) −*x*+2, −*y*, −*z*+1.
